# Inhibition of *Perilla frutescens* Essential Oil on Pellicle Formation of *Candida tropicalis* and *Pichia kluyveri* and Its Effect on Volatile Compounds in Sichuan Pickles

**DOI:** 10.3390/foods12081593

**Published:** 2023-04-09

**Authors:** Ting Cai, Pei Shi, Shan Zhang, Wenliang Xiang, Junyu Liu, Zixi Lin, Jie Tang

**Affiliations:** 1 School of Food and Bioengineering, Xihua University, Chengdu 610039, China; 2 Key Laboratory of Food Microbiology of Sichuan, Xihua University, Chengdu 610039, China; 3 Chongqing Key Laboratory of Speciality Food Co-Built by Sichuan and Chongqing, Xihua University, Chengdu 610039, China

**Keywords:** *Perilla frutescens* essential oil, pellicle formation, *Candida tropicalis*, *Pichia kluyveri*, volatile compounds, Sichuan pickles

## Abstract

Pellicle formation is the most typical characteristic of deteriorating fermented vegetable products. *Perilla frutescens* essential oil (PEO) is widely used as a useful natural preservative. However, few studies have addressed the antifungal activity and mechanism of PEO in pellicle formation microorganisms, and it is still unclear whether it can inhibit pellicle formation and affect its volatile compounds in Sichuan pickles. The current study showed that PEO can inhibit pellicle formation during fermentation of Sichuan pickles as it had significant antifungal activity against the pellicle formation microorganisms *Candida tropicalis* SH1 and *Pichia kluyveri* SH2. The minimum inhibitory concentration (MIC) of PEO against *C. tropicalis* SH1 and *P. kluyveri* SH2 was determined to be 0.4 μL/mL, and the minimum fungicidal concentrations (MFCs) were 1.6 μL/mL and 0.8 μL/mL, respectively. The antifungal mechanism was activated as a result of damage to the cell membrane, an increase in the cell permeability, a decrease in the mitochondrial membrane potential, and the inhibition of ATPase activity. Meanwhile, the addition of PEO to Sichuan pickles can enrich the profiles of volatile compounds during fermentation, including limonene, myrcene, 1,8-cineole, linalool, perilla ketone, heptanal, hexanal, α-thujone and β-terpineol and thus improve the overall sensory acceptability. These results indicated that PEO has the potential to be used as a novel food preservative to control pellicle formation in fermented vegetables.

## 1. Introduction

Vegetable fermentation is one of the oldest known preservation methods for various seasonal vegetables and it plays a key role in improving the flavor, appetite, and nutritional value of products [1,2]. Sichuan pickles, as a typical representative of the art of traditional vegetable fermentation, and their unique flavor and potential health benefits have made them widely consumed in Asia, especially China. The industrial-scale production output of Sichuan pickles reached 4.8 million tons and was valued at over USD 6.1 billion dollars in 2021. However, spoilage phenomenon of Sichuan pickles occurs occasionally, which include discoloration, pellicle formation, tissue softening, and so on [3]. Pellicle formation is the most typical characteristic that occurs during the deterioration of fermented vegetable products, and the genera *Pichia* and *Candida* have been reported to be the principal players in the pellicle formation of Sichuan pickles [4,5,6,7]. Pellicles can significantly reduce the sensory quality of Sichuan pickles by softening the vegetables’ texture and producing off-flavors. These cause a potential food safety issue that leads to significant economic losses [8,9]. Therefore, inhibiting pellicle formation has consistently been an important problem that urgently needs to be addressed within the Sichuan pickle industry.

Plant-derived essential oils are widely used as natural preservatives in the food industry due to their outstanding biological activities. Most importantly, a large body of research has shown that most essential oils derived from plants are generally recognized as safe (GRAS) [10,11]. *Perilla frutescens* (L.) Britton belongs the family Lamiaceae and is widely distributed in east Asian countries, such as Japan, China, Korea, and Vietnam. Its leaves are widely used as a culinary herb and spice because of their aromatic and pleasant colors. The leaves are also used as a preservative in pickled foodstuffs (vegetables and fruits) [12,13]. PEO derived from *Perilla frutescens* leaves has generated a growing amount of interest due to its outstanding bioactivity in aromatic medicinal plants, including its antioxidant, antimicrobial, and anti-inflammatory properties [12,14,15]. Studies have found that the antimicrobial function of PEO is mainly due to its bioactive secondary metabolites, such as perillaldehyde, limonene, perilla ketone, and so on, which can significantly inhibit the growth of food-borne pathogenic strains [13,16,17]. However, to date, there are few studies on the inhibition of PEO on pellicle formation microorganisms and the effect of PEO as a preservative on the flavor of Sichuan pickles is still unclear.

Therefore, this study focused on assessing the inhibition of PEO on the pellicle formation microorganisms *C. tropicalis* SH1 and *P. kluyveri* SH2 in relation to Sichuan pickles. An analysis of the effect of PEO as a preservative on the volatile compounds during the fermentation of Sichuan pickles was carried out. The results could provide a reliable theoretical and scientific basis for the development of highly efficient and low toxicity bacteriostatic agents of natural extracts.

## 2. Materials and Methods

### 2.1. Reagents

*Perilla frutescens* leaves were purchased from Haozhou Xinrongtang Co., Ltd. (Haozhou, China). Fluconazole (32–320 μM) was purchased from Sigma-Aldrich. Rhodamine 123 was purchased from Beijing Solaibao Technology Co., Ltd. (Beijing, China).

### 2.2. Yeast Strains and Culture Conditions

*P. kluyveri* SH2 (CGMCC 2.6449) and *C. tropicalis* SH1 (CGMCC 2.6448) were isolated from the Sichuan pickle’s pellicle. The strains were activated and cultured in potato dextrose broth (PDB) as indicated by Yu et al. [17]. Then, the cells were cultured when they reached the logarithmic growth stage and centrifugated at 1750× *g* for 1 min and then adjusted to 10^6^ CFU/mL with phosphate buffered saline (PBS) for subsequent tests.

### 2.3. Isolation of Perilla frutescens Essential Oils

The essential oil was isolated according to the method of the 2015 edition of Chinese Pharmacopoeia [18]. The 10 g homogenized leaves were placed in a 1 L round-bottomed flask filled with 500 mL distilled water and boiled for 3 h using a Clevenger-type apparatus. The essential oil was distilled following water vapor and collected after reflux condensation at 25 °C. Then, it was stored in dark vials sealed with Teflon-faced septa (Supelco, Bellefonte, CA, USA) and kept at 4 °C.

### 2.4. GC-MS Analysis of PEO

The GC–MS analysis was carried out using an Agilent Technologies 6890 N gas chromatograph coupled to an Agilent Technologies DB—5MS capillary column (30 m × 0.25 mm, 0.25 μm film thickness) according to Ahmed et al. [13], working with the following temperature program: initial temperature 55 °C for 4 min; heating by a rate of 10 °C/min up to 130 °C; then by a rate of 6 °C/min up to 240 °C; the final temperature was maintained for 10 min; injector and detector temperatures: 250 °C; carrier gas: helium (constant flow rate of 1 μL/min); the split ratio was adjusted to 1:30; injection volume was 0.2 μL. The acquisition mass range was 40–400 *m*/*z* and the ionization voltage were 70 eV. The constituents were tentatively identified by matching the mass spectrum with the NIST5 spectrum database and verified by comparison using C7-C40 n-alkanes.

### 2.5. Antifungal Activity of PEO against P. kluyveri and C. tropicalis

The antifungal activity of PEO against *P. kluyveri* SH2 and *C. tropicalis* SH1was evaluated using the MIC and MFC via the tube dilution method as described by Hu et al. [10]. The PEO was diluted to serial concentrations ranging from 6.4 to 0.1 μL/mL with a Tween-20 (0.1%) solution. Subsequently, a spore solution of *C. tropicalis* SH1 and *P. kluyveri* SH2 was suspended in PDB medium in 96-well plates. Their growth was measured by spectrophotometry at a 600 nm wavelength following incubation at a 30 °C for 24, 48, 72, and 96 h. Simultaneously, the sample that with fluconazole (32 μM) and the sample containing only the PDB medium were considered the positive control and negative controls, respectively. Compared to the control, the minimum PEO concentration that did not allow for visible growth was MIC. MFC was considered to have the lowest PEO concentration inhibiting fungal growth [19].

### 2.6. Antifungal Mechanism

#### 2.6.1. Morphological Characteristics

The change in cell morphology was observed through scanning electron microscopy (SEM) (Zeiss, Jena, Germany) as described by Yu et al. [17]. MIC PEO was added to the medium of *C. tropicalis* and *P. kluyveri* growing in the logarithmic phase. After incubation at 30 °C for 4 h at 200 revolutions per minute continuous agitation, the cell suspensions were harvested by centrifugation at 4000 revolutions per minute for 5 min and washed twice by phosphate buffer saline (PBS, pH 7.4). The diluted pellets were adhered to a slide and fixed in 2.5% (*v*/*v*) glutaraldehyde for 5 h at 4 °C. Then, the cells on the slide were successively dehydrated in 20, 40, 60, 80, and 100% ethanol several times. Subsequently, the slides with cells were freeze-dried at −40 °C for 12 h and then sputter-coated with gold. The cells from PDB without PEO were used as a control. The cell morphological changes were observed in an FEI Inspect F50 scanning electron microscope (Inspect F50; FEI, Hillsboro, OR, USA).

#### 2.6.2. Mitochondrial Membrane Potential

The determination of the mitochondrial membrane potential through fluorescent JC-1 probes (5,5′, 6,6′-tetrachloro-1,1′, 3,3′-tetraethyl-imicarbanine iodization method) was determined according to the method described by Kang et al. [20]. Briefly, the strains were incubated and adjusted to 1 × 10^6^ CFU mL-1 with the PDB medium. After adding MIC PEO and being incubated at 30 °C for 3 h, the JC-1 dye was added to 10 μg/mL and incubated again in the dark for 16 min. The solution was centrifuged at 1750× *g* for 1 min and then resuspended twice with PBS (pH 7.4) to completely remove the extracellular JC-1. The PBS (pH 7.4) treatment was the control. The fluorescent intensity of green signal (JC-1 monomer, 485 nm excitation and 535 nm emission) and red signal (JC-1 aggregate, 550 nm excitation and 600 nm emission) were measured, respectively, with a Tecan Infinite M1000 Pro reader. The mitochondrial membrane potential was reflected by the intensity ratio of the red to green fluorescence.

#### 2.6.3. Cell Membrane Permeability

The relative conductivity was used to represent the change in the cell membrane permeability of *C. tropicalis* SH1 and *P. kluyveri* SH2. It was characterized by the electrolyte leakage from cells into the culture medium referring to the method of Yu et al. [17]. The cells without MIC PEO were considered as a control.

#### 2.6.4. Cellular Adenosine Triphosphatase (ATPase) Activity

The suspension of *C. tropicalis* SH1 and *P. kluyveri* SH2 in PDB was inoculated at 30 °C for 3 h after treatment with the MIC PEO and then centrifuged at 1750× *g* for 5 min and resuspended in 10 mL of PBS (pH 7.4). Then, the sample was lysed in EDTA buffer (2 mM, pH 9.0) by intermittent ultrasound at 40 s ultrasound and 20 s intervals for 2 min under 450 W power in an ice bath. The inhibitory effect of PEO on the ATPase of *C. tropicalis* and *P. kluyveri* was accomplished using an ATPase assay kit (Beijing Solarbio Technology Co., Ltd., Beijing, China).

### 2.7. Effects of PEO on the Quality of Sichuan Pickle

#### 2.7.1. Pickles Preparation

Sichuan pickles were prepared using fresh white radish and following the traditional fermentation method [1]. PEO was added into a pottery jar in order to inhibit pellicle formation (PEO group: 5.0 μg/mL), and the pottery jar without PEO was considered the blank group (CON group). The jars were placed in an incubator at 25 °C for 7 days (the lid was opened for 5 min every day). Then, 20 mL salt brine from each treatment (n = 3) and 10 g of white radish samples were collected for analysis.

#### 2.7.2. Analysis of Physicochemical Indexes

The physicochemical indexes, including total sugar, reducing sugar, and pH value were determined through the salt brine sample. The total and reducing sugars were calculated through the dinitrosalicylic acid method with a UV-Vis spectrophotometer (V-570; Jasco Co., Ltd., Tokyo, Japan) [21]. The pH value was measured with a pH meter (PHS-3C; Fangzhou Technology, Chengdu, China). A 10 g amount of white radish was homogenized for 4 min before being decontaminated and defatted with 20 mL of 0.42 mol/L ZnSO4 precipitation and then filtered. Nitrite levels were determined through the Griess reaction method [22].

#### 2.7.3. Analysis of the Volatile Compounds

Headspace solid phase microextraction (HS–SPME) combined with gas chromatography–mass spectrometry (GC–MS) was employed to extract and quantitatively analyze the volatile compounds from Sichuan pickles as described by Xiang et al. [1]. The 5 mL sample was placed in a 15 mL SPME glass vial together with 2 g NaCl and 10 μL of the internal standard 2-octanol (30 mg/L in absolute ethanol). Then, a 75 μm Carboxen/PDMS StableFlex fiber (Supelco, Bellefonte, PA, USA) was placed in the SPME device, which was inserted into the vial and extracted for 40 min at 50 °C with magnetic stirring. Then, it was desorbed for 7 min at 250 °C in the GC inlet with the automatic autosampler. The separation of volatile compounds was performed with a DB—5MS capillary column (30 m × 0.25 mm, 0.25 μm film thicknesses, Agilent Technologies, Inc, Palo Alto, CA, USA). Mass parameters were obtained by electron impact ionization at 70 eV, and the ion source and transfer line temperature were held at 230 °C and 250 °C, respectively. The mass range was 40–400 *m*/*z*. The constituents were tentatively identified by matching the mass spectrum with the NIST5 spectrum database and verified by comparison using C7-C40 n-alkanes.

#### 2.7.4. Sensory Analysis

The sensory evaluation of Sichuan pickles was carried out according to the method described by Xiang et al. [1]. A total of eight sensory indicators associated with pickle quality were selected, including appearance (color and form), smell (vegetable aroma, composite aroma, and corruption smell), taste, and texture (astringency, sour, and brittleness). The 10 experts (five males and five females) with extensive experience in the field of Sichuan pickles carried out the sensory evaluation.

### 2.8. Statistical Analyses

All analyses were repeated in triplicate and the data were presented as the mean values ± standard deviation. The significance of the differences between the samples was determined through an ANOVA with Tukey’s post hoc test using SPSS, version 20.0 (IBM Corp., Armonk, NY, USA). The differences in the volatile compounds between the groups were visualized through a heat map and cluster diagrams using TBtools v1.098, orthogonal projections analysis (OPLS-DA) with SIMCA software (version 14.1) (Umetrics, Sweden). Then, the important volatile compounds with variable importance for the projection (VIP) > 1.00 were visualized using Circos software (http://mkweb.bcgsc.ca/tableviewer/visualize/ accessed on 12 December 2022). 

## 3. Results

### 3.1. The Inhibitory Effect of PEO on Pellicle Formation

**Figure 1:**
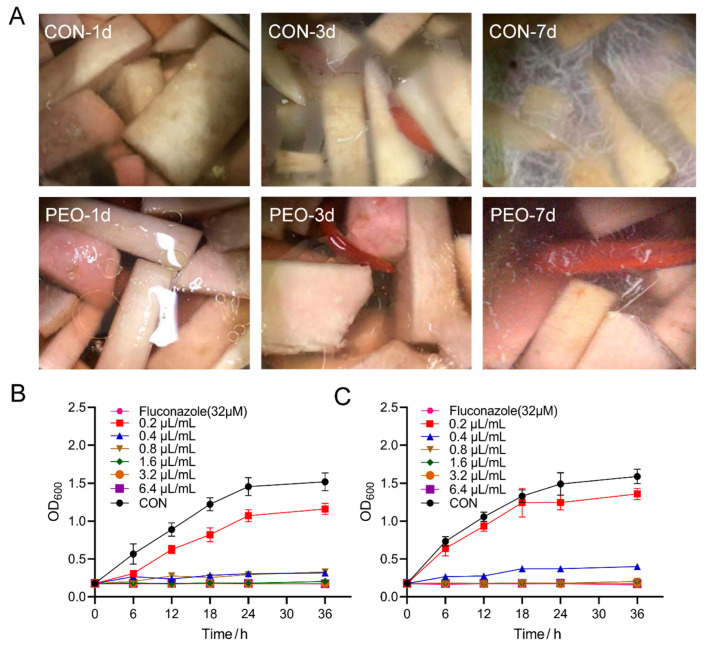
Inhibitory effect of PEO on pellicle formation and its antifungal activity. (**A**): the inhibition of PEO on pellicle formation of Sichuan pickles during fermentation; antifungal activity of PEO against *C. tropicalis* SH1 (**B**) and *P. kluyveri* SH2 (**C**).

Pellicle formation in Sichuan pickles is the main cause for the deterioration of their sensory quality, which typically appears as an unpleasant odor, a softer texture, and an increase in the thickness of the skin; this leads to great economic losses [7,23]. In the present study, *C. tropicalis* SH1 and *P. kluyveri* SH2 isolated from Sichuan pickles, had a certain ability to form pellicles (Figure S1). As alternative agents for food preservation, the addition of PEO could significantly inhibit pellicle formation during the fermentation process (Figure 1A). The values of the MIC and MFC quantitatively assessed the antifungal activity of PEO. The imperceptible change in the OD values of *C. tropicalis* SH1 and *P. kluyveri* SH2 after exposure to PEO at various time points of the experiment indicates its fungicidal effects. The MIC of PEO against *C. tropicalis* SH1 and *P. kluyveri* SH2 was 0.4 μL/mL, while the MFC was 1.6 μL/mL and 0.8 μL/mL, respectively (Figure 1B, C). Only when the PEO concentration was greater than 1.6 μL/mL, *P. kluyveri* SH2 could be completely inhibited, and the OD curve almost coincided with that of the CON group (Figure 1C). To analyze the major chemical components of PEO, 17 compounds with relatively high contents of PEO were identified by GC–MS and were characterized by the predominance of linalool (51.62%), limonene (11.62%), perilla ketone (5.11%), 1,8-cineole (4.68%), myrcene (1.93%), α-pinene (1.98%), and β-pinene (1.65%) (Table S1). The current results show that the main components of PEO are monoterpenoids and their oxides, including limonene, linalool, and myrcene.

### 3.2. Antifungal Mechanism of PEO against Pellicle Formation

PEO has a better application prospect in food preservation because its outstanding biological activity is non-toxic or exerts very low toxicity [10]. However, the antifungal activity and specific molecular mechanisms of PEO against pellicle formation microorganisms are rarely reported. Here, the mechanism of PEO’s activity against the strains was explored from several aspects, including cell morphology, membrane permeability, mitochondrial membrane potential, and ATP production.

**Figure 2:**
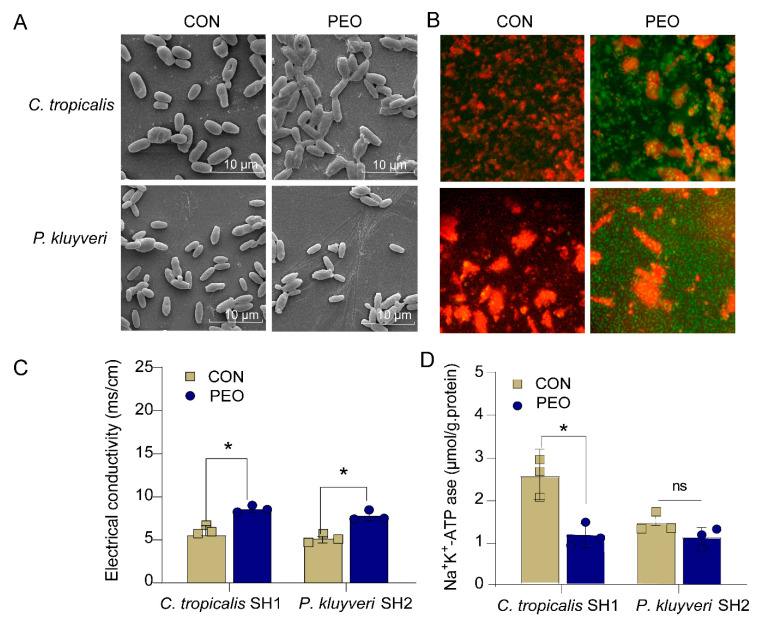
Antifungal mechanism of PEO against *C. tropicalis* SH1 and *P. kluyveri* SH2. (**A**): The cell morphology treatment with the MIC PEO of *C. tropicalis* SH1 and *P. kluyveri* SH2 by SEM; (**B**): fluorescence intensity images mitochondrial membrane potential treatment with PEO of *C. tropicalis* SH1 and *P. kluyveri* SH2; (**C**): effects of PEO on the cell membrane permeability of *C. tropicalis* SH1 and *P. kluyveri* SH2; (**D**): effects of PEO on Na^+^ K^+^-ATPase of *C. tropicalis* SH1 and *P. kluyveri* SH2. The * indicates the significant level: *, *p* < 0.05; ns, *p* > 0.05.

Usually, the morphological alteration of the cell occurs after treatment with antimicrobial agents [24]. The SEM results indicated significant differences in the cell morphology between the samples of *C. tropicalis* SH1 and *P. kluyveri* SH2 treated with PEO and the samples that were left untreated. The cells in the CON groups retained a normal cell morphology, full of sharp edges. On the contrary, *C. tropicalis* SH1 cells treated with PEO had what appeared to be deformed surfaces, the cells displayed shrinkage and their surfaces were wrapped in a transparent membrane. In parallel, *P. kluyveri* SH2 cells also had significantly deformed surfaces and the multiple cells were gathered into a mass (Figure 2A). In the present study, relative electrical conductivity was employed to evaluate the cell membrane permeability. PEO is known to disrupt the normal cell membrane permeability of *C. tropicalis* SH1 and *P. kluyveri* SH2, and the electrical conductivity was observed to be significantly higher in those cell groups than in the CON groups (*p* < 0.05) (Figure 2C). 

The mitochondrial membrane potential (MMP, ∆Ψm) is a necessary prerequisite for mitochondria to function, and a decrease in ∆Ψm is generally considered to be a common phenomenon before any morphological changes occur in cells [25,26]. The fluorescence intensity images showed that PEO can destroy the mitochondrial membrane potential of *C. tropicalis* SH1 and *P. kluyveri* SH2. The red fluorescence intensity was significantly reduced, while the green fluorescence intensity was enhanced (Figure 2B). Therefore, ∆Ψm was significantly decreased (Figure S2) (*p* < 0.05). The results suggest that depolarization of the cell membrane and an imbalance in ion motion occurred when *C. tropicalis* SH1 and *P. kluyveri* SH2 were exposed to PEO. In addition, the results of the intracellular Na^+^/K^+^-ATPase activity showed that ATP production had a 40% reduction in the sample *C. tropicalis* SH1 treated with PEO (Figure 2D) (*p* < 0.05), while the decrease in ATP production was not significant for *P. kluyveri* SH2 (*p* > 0.05). These results indicate that PEO has a good potential for preventing pellicle formation in Sichuan pickles as it prevents the pellicle formation microorganisms from damaging the cell membrane and changing the cell permeability. It also prevents it from inhibiting the ATPase activity and destroying the mitochondrial membrane potential.

### 3.3. Effect of the Physicochemical Indexes during Fermentation

**Figure 3:**
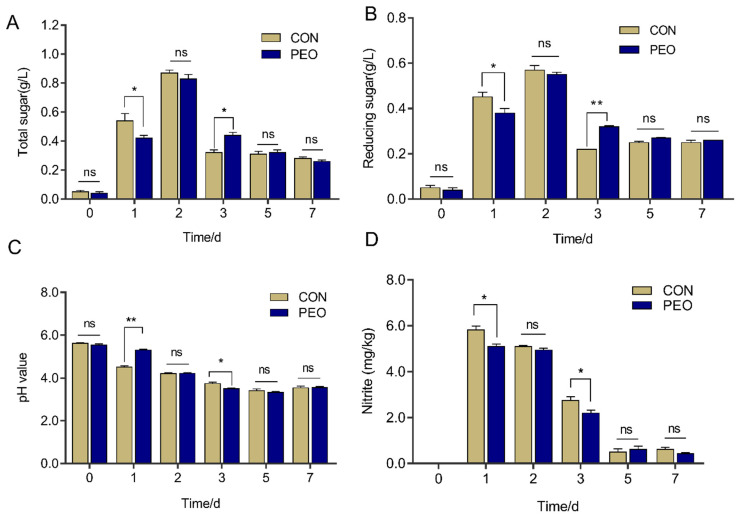
The concentration of total sugar (**A**), reducing sugar (**B**), pH value (**C**), and nitrite (**D**) in CON and PEO during fermentation. The * indicates the significant level: *, *p* < 0.05; **, *p* < 0.01; ns, *p* > 0.05.

In the present study, the concentrations of the total sugar and reducing sugar showed similar change trends in the CON and PEO groups. At day 1, the concentrations of the total sugar and reducing sugar were significantly lower in PEO than in CON (*p* < 0.05). With the continuous process of fermentation, the concentrations of the total sugar and reducing sugar in PEO and CON reached peak values at day 2, and then showed a downward trend between days 2 and 7. These results were not significantly different from those obtained in the CON groups between days 5 and 7 (*p* > 0.05) (Figure 3A,B). However, the pH values showed significantly different changes from those of the total and reducing sugars. The pH value was significantly higher in PEO than in CON at day 1 (*p* < 0.01), and with the continuing fermentation, the pH value of PEO decreased continuously, and was significantly lower than that of CON at day 3 (*p* < 0.05). The pH value remained at around 3.5 for the PEO and CON groups between days 5 and 7 (Figure 3C). The nitrite concentrations in all the test samples were lower than the maximum value of 20 mg/kg for the fermentation of vegetables in China. During the whole fermentation process, the nitrite concentrations were reduced, and the content of nitrite in PEO was significantly lower than that in the CON group between days 1 and 3 (Figure 3D). Therefore, the changes in the physicochemical indicators suggested that the addition of PEO changed the metabolic activity of Sichuan pickles during the fermentation process to a certain extent, but it has little effect on the fermented products.

### 3.4. Changes of Volatile Components

**Figure 4:**
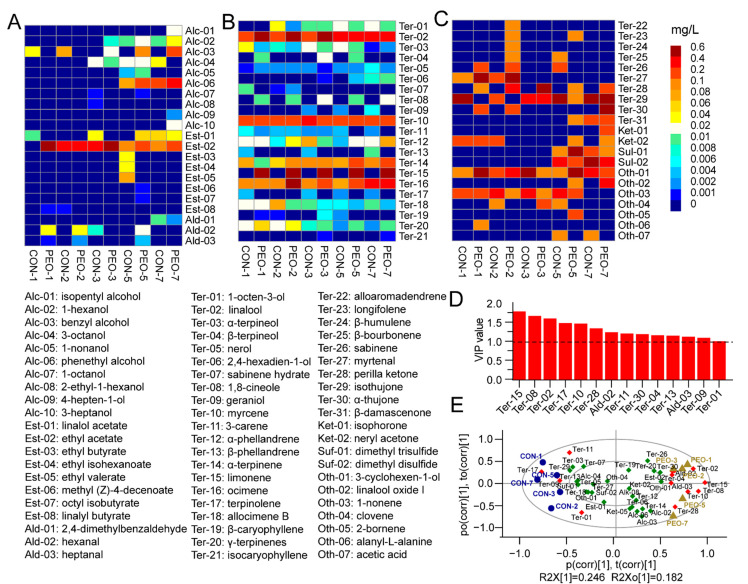
The distribution heat map and cluster diagrams of the volatile compounds in CON and PEO during the fermentation. (**A**): the heat of alcohols, esters, and aldehydes; (**B**): the heat of 21 terpenoids; (**C**): the heat of other categories (some terpenoids, sulfides, ketones, and other compounds); (**D**): the VIP value of the important volatile compound variables (VIP > 1.0) in PEO and CON; (**E**): the distribution of the differential metabolites in PEO and CON (green: 0.75 < VIP < 1.0; red: VIP > 1.0).

Volatile composition is one of the most important characteristics of traditional fermented food products and one of the main factors determining food quality [27]. A total of 63 main volatile compounds were detected in the CON and PEO groups following fermentation for 7 days, including 31 terpenoids, 10 alcohols, 8 esters, 3 aldehydes, 2 ketones, 2 sulfides, and 7 others (Figure 4A–C). Furthermore, 44 and 55 volatile compounds were found in the CON and PEO groups during fermentation, respectively (Table S2). Evidently, PEO markedly enriched the composition and content of the volatile components (Figure 4 and Table S2). During fermentation, the total content of total volatile components in PEO was significantly higher than that in CON at days 1, 2, 3, 5, and 7, including total terpenoids, ketones, and esters at 1, 2, 3, and 7 d. Among the total volatile components, terpenoids, esters, and alcohols contributed more than other types of compounds during fermentation.

**Figure 5:**
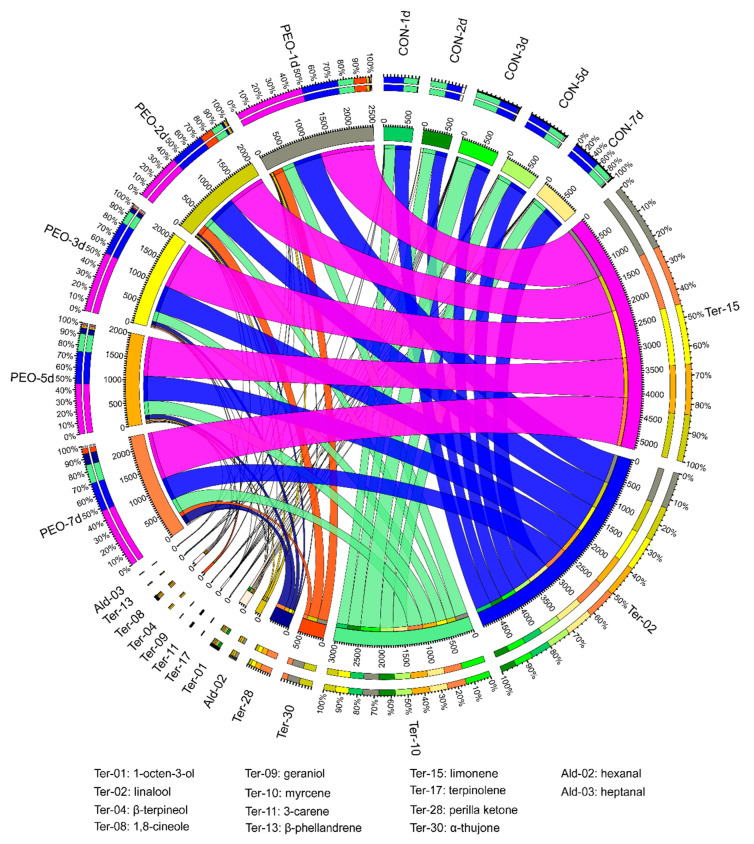
The distribution of VIP > 1 volatile compounds in Sichuan pickles for CON and PEO during fermentation. The data from the average value of three independent experiments were visualized via Circos software (http://circos.ca/ accessed on 12 December 2022). The bar length on the outer ring represents the percentage of each volatile compound in each sample. The bar length on the inter-ring represents the content (μg/L) of each volatile compound in each sample.

The OPLS-DA analysis of 39 different metabolites further indicated that CON and PEO could be easily separated (Figure 4E). Among them, 14 volatile compounds had a variable importance for the projection (VIP) greater than 1.0 (Figure 4D); 5 volatile compounds (VIP > 1.0) were the important variables in CON, including terpinolene, 3-carene, 1-octen-3-ol, geraniol, and β-phellandrene; and 9 volatile compounds (VIP > 1.0) were the important variables in PEO, including limonene, myrcene, 1,8-cineole, linalool, perilla ketone, heptanal, hexanal, α-thujone, and β-terpineol (Figure 4E and Figure 5). The limonene, 1,8-cineole and perilla ketone were found only in PEO and were also determined through GC–MS to be important components of PEO (Figure 5 and Table S1), which suggested that PEO can enrich the profiles of volatile compounds during fermentation.

### 3.5. Sensorial Analysis

**Figure 6:**
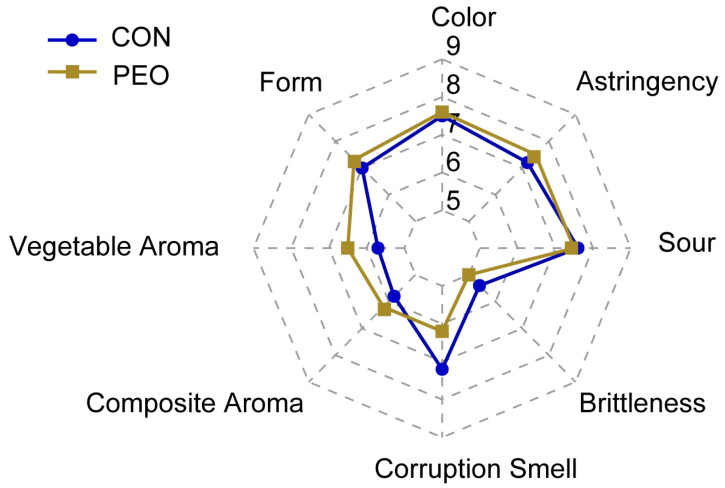
The sensory profile of the organoleptic attributes of CON and PEO fermented for 7 days. The date is the average value of three independent experiments.

In summary, the Sichuan pickles in the PEO group had a more intense aroma and harmonious taste than those in the CON group (Figure 6). The Sichuan pickles in the PEO group had a higher descriptor score in form (7.3), vegetable aroma (6.5), composite aroma (6.2), and astringency (7.5) than those in the CON (7.0, 5.7, 5.8, and 7.2). The PEO pickles had a lower score in corruption (6.2) and brittleness (5.0) than the CON pickles (7.2 and 5.4), and both had a similar score for color and taste (7.5–7.6). The results showed that PEO was enriched with more plentiful alcohols and esters to improve the vegetable and composite aromas. Meanwhile, PEO can effectively inhibit the pellicle formation and reduce the corruption and brittleness of Sichuan pickles. Although the scores for the sensorial indexes varied for Sichuan pickles in the CON and PEO groups, the overall sensory characteristics were highest for those in the PEO group.

## 4. Discussion

The characteristics of spontaneous fermentation and the lack of secondary sterilization make fermented vegetables susceptible to spoilage microorganisms. Pellicle formation is the most typical characteristic in the aerobic deterioration of fermented vegetable products [8,28]. The pellicle itself is not toxic, but some of the pathogenic or spoilage microbes may attach therein and increase toxicity, and in severe cases, it will affect the overall flavor of fermented vegetables. Therefore, inhibiting pellicle formation during the fermentation process is of great significance to improve the quality and safety of fermented vegetables. Previous studies have obtained a better understanding of the composition of pellicle formation and its functional characteristics related to the microbial community in Sichuan pickles during the fermentation system, but to our knowledge, little is known about prevention and control strategies.

PEO has been shown to be an effective and reliable natural food antimicrobial agent [12,15]. Pellicle formation is considered to be a sign of deterioration and is mainly composed of Pichia, *Debaryomyces,* and *Candida* [8,17,28]. The current study demonstrates that the PEO can inhibit pellicle formation in Sichuan pickles and has a significant antifungal activity against *C. tropicalis* SH1 and *P. kluyveri* SH2 (Figure 1). The main components of PEO are limonene, linalool, and perilla ketone, as determined through GC–MS. Limonene is a common artificial monocyclic monoterpene, which is a potential antimicrobial component in plant essential oils [17]. Studies have demonstrated that limonene has good inhibitory effects for *Colletotrichum falcatum*, *Staphylococcus aureus*, *Aspergillus niger,* and *Listeria monocytogenes* [29]. Wang et al. also reported that perilla ketones isolated from perilla essential oil are important antifungal components [30]. Tian et al. reported that perillaldehyde was the main antifungal component in PEO [31], but it was not detected in the current study. This might be due to differences in variety, growth period, and the growth environment of *Perilla frutescens* that can lead to the changes in the types and proportions of essential oil components and result in differences in the active components [32]. However, some scholars are now finding that the influence of trace components in plant essential oils on antifungal activity are more important than that of the main components in plant essential oils. The combination of the main components and other trace components with weak activities may achieve a synergistic effect [33], which make plant essential oils have greater antifungal activities than the main components mixed with them.

The current study has demonstrated that the MIC PEO can penetrate the cell wall and destroy the membrane and increase the membrane permeability (Figure 2A,C), which may lead to the leakage of intracellular proteins; meanwhile, PEO destroys the mitochondrial membrane potential of *C. tropicalis* SH1 and *P. kluyveri* SH2 (Figure 2B and Figure S2) and this leads to less ATP synthesis for *C. tropicalis* SH1 (Figure 2D). The mitochondrial membrane potential is an important element of ion motive force. It is involved in the generation of ATP and metabolic activities in cells and the decrease of the mitochondrial membrane potential is the characteristic mark of apoptosis [34,35]. PEO may inhibit pellicle formation in Sichuan pickles by affecting the cell membrane, increasing membrane permeability and damaging the mitochondrial membrane potential. Hu et al. also reported that PEO can inhibit *Aspergillus flavus* growth through leakage of macromolecules within the cells, it can inhibit ATPase activity and damage mitochondrial membrane potential [10], but there are also studies that show that the antifungal action of PEO disrupts the energy metabolism and defense function [36]. Therefore, the mechanism of PEO against *C. tropicalis* SH1 and *P. kluyveri* SH2 needs to be further explored through transcriptomic and proteomic analyses.

In general, the addition of PEO changes the metabolic activity of Sichuan pickles during the fermentation process to a certain extent, but it has little effect on the final fermented products (Figure 3). However, it was observed that the concentrations and types of volatile components in the CON and PEO groups were significantly different during fermentation. The important volatile components, including the β-terpineol, 1, 8-cineole, limonene, perilla ketone, hexanal, heptanal, and α-thujone were only detected in PEO; the concentrations of 3-carene were significantly lower; and myrcene and linalool were higher in PEO than CON (Figure 4 and Figure 5). The 1,8-cineole, limonene, and perilla ketone form the major chemical composition of PEO (Table S1). As a kind of saturated monoterpene, 1,8-cineole has major pharmacological properties, such as anti-inflammatory and antioxidative properties [36], and it is extracted mainly from the essential oils of plants, including Fructus *Alpiniae zerumbet*, *Salvia lavandulifolia Vahl*, and *Eucalyptus*, which are often added to fragrances, cosmetics, or perfumes due to their pleasant aromas and taste [37]. Bassoil et al. reported that perilla ketone was responsible for the flavors of *Perilla frutescens* and it is one of the most important components in the aroma of the leaves of the Korean type of *P. frutescens* *(kennip)* [38]. As one of the active components of essential oils, it showed no inhibitory effect on white film in this study, possibly because the target of its antimicrobial spectrum was usually bacteria rather than fungi [31]. Studies reported that α-thujone is a monoterpenoid isolated from the essential oil of *Ajania 12ruticulose* and has effective antitumor activities [39,40]. Additionally, hexanal and heptanal are found naturally in *Perilla*, California lemon, and rose, which can improve the fermented food quality by endowing its fragrance and fruity notes, and a long-lingering nut-like taste [41,42]. Terpineol and linalool are alcohols and these alcohols not only give Sichuan pickles a soft flavor, but also cooperate or react with phenylacetic acid, phenyl acetate, phenyl ethanol, and other substances generated by lactic acid bacteria and the metabolism of amino acids, therefore, presenting a more pleasant flavor or generating more complex flavor substances [1]. Linalool, for instance, makes an important contribution to the flavor of pickled ginger and radish. In addition, 51.62% of PEO is linalool (Table S1), so the content of PEO is significantly higher than CON between days 3 and 7 during fermentation. The acidification degree of Sichuan pickles increased between days 5 and 7 (Figure 3C). It also led to the oxidation of linalool and the formation of linalool oxide I with black tea aroma [43], which was detected in a small amount of PEO in the later stage of fermentation. There were two sulfides that were detected between days 5 and 7 during fermentation, dimethyl disulfide and dimethyl trisulfide, which are trace components in many fermented products and bring a typical sulfur odor to the samples and has a significant negative effect on the fermentation flavor. Ao et al. reported that dimethyl sulfide was the main pungent odor in spoiled Sichuan pickles [44], and the content of dimethyl sulfide in the PEO group is significantly lower than that in the CON group, which reduces the odor and improves the overall acceptability of the pickles (Figure 6).

## 5. Conclusions

This study revealed that PEO can effectively inhibit pellicle formation in the fermentation process of Sichuan pickles and has a significant antifungal activity against the pellicle formation microorganisms *C. tropicalis* SH1 and *P. kluyveri* SH2. The antifungal mechanism is activated as a result of damage to the cell membrane, an increase in the cell permeability, a decrease in the mitochondrial membrane potential, and the inhibition of ATPase activity. Meanwhile, the addition of PEO to Sichuan pickles can enrich the profiles of volatile compounds and improve the acceptability of the products. Further studies involving transcriptomics will be conducted in order to analyze in more depth the mechanism of PEO in pellicle inhibition.

## Data Availability

Raw data obtained in this study are available from the corresponding author upon reasonable request.

## Supplementary Materials

The following supporting information can be downloaded at: https://www.mdpi.com/article/10.3390/foods12081593/s1, Figure S1: The pellicles formation of *C. tropicalis* SH1 and *P. kluyveri* SH2, Figure S2: Effects of PEO on mitochondrial membrane potential of *C. tropicalis* SH1 and *P. kluyveri* SH2, Table S1: Major chemical composition of PEO, Table S2: The content (mg/L) of each volatile compound in CON and PEO samples during fermentation.
