# Supplementary material for: Inhibition of Perilla frutescens Essential Oil on Pellicle Formation of Candida tropicalis and Pichia kluyveri and Its Effect on Volatile Compounds in Sichuan Pickles
Source: Foods. 2023 Apr 9;12(8):1593. doi: 10.3390/foods12081593 (PMC10137390; doi:10.3390/foods12081593)
Supplement: Supplementary file 1 [file foods-12-01593-s001.zip › foods-2251049-SI.pdf]

Supplementary figure legends

Figure S1. The pellicles formation of *C. tropicalis* SH1 and *P. kluyveri* SH2.

Figure S2. Effects of PEO on mitochondrial membrane potential of *C. tropicalis* SH1 and *P. kluyveri* SH2. Bars represent the standard deviation (n = 3). Dates were assessed by ANOVA with Tukey's post hoc test. The * indicated significant level: *, $p < 0.05$.

Supplementary table notes

Table S1. Major chemical composition of PEO.

Table S2. The content (mg/L) of each volatile compound in CON and PEO samples during fermentation.

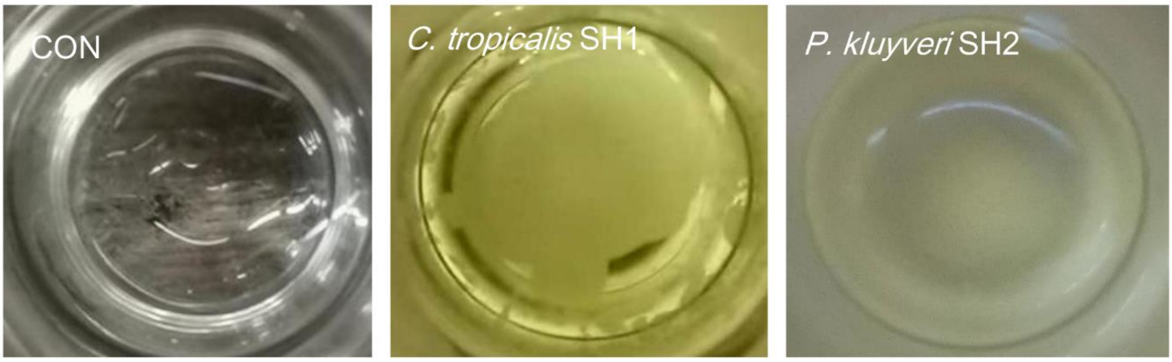

Figure S1. The pellicles formation of *C. tropicalis* SH1 and *P. kluyveri* SH2

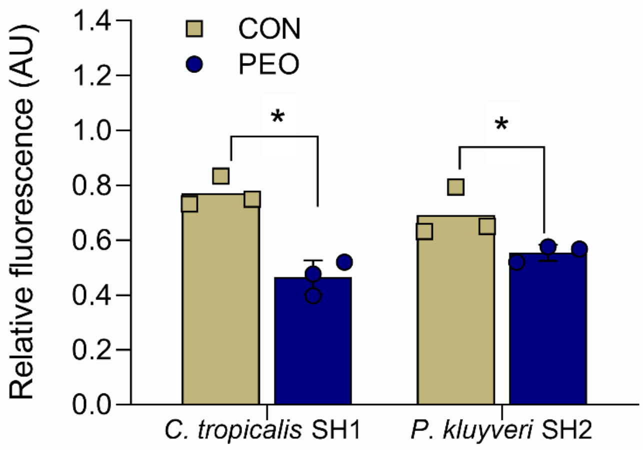

Figure S2. Effects of PEO on mitochondrial membrane potential of *C. tropicalis* SH1 and *P. kluyveri* SH2. Bars represent the standard deviation (n = 3). Dates were assessed by ANOVA with Tukey's post hoc test. The * indicated significant level: *, $p < 0.05$.

Table S1. Major chemical composition of PEO

NO	Compounds	Composition (%) [a]	RI[b]
1	2,4-dimethyl-1-heptene	0.65	819
2	n-nonane	0.09	919
3	α-pinene	1.98	948
4	camphene	0.18	952
5	4-octanone	0.13	962
6	sabinene	0.32	968
7	p-mentha-1(7),2-diene	0.75	974
8	β-pinene	1.65	983
9	myrcene	1.93	998
10	1,8-cineole	4.68	1011
11	o-cymene	1.01	1025
12	limonene	11.62	1089
13	2-nonen-1-ol	0.22	1167
14	perilla ketone	5.11	1211
15	linalool	51.62	1272
16	eugenol	0.02	1342
17	α-terpineol	0.42	1590

a: Percentage based on FID peak area normalization. b: Retention indices.

Table S2. The content of each volatile compound in CON and PEO samples during fermentation (mg/L)

Volatile compounds	Fermentation time (d)									
	1		2		3		5		7	
	CON	PEO	CON	PEO	CON	PEO	CON	PEO	CON	PEO
**Alcohols**										
isopentyl alcohol	nd	nd	nd	nd	nd	nd	nd	0.001±0.001	nd	0.027±0.002
1-hexanol	nd	nd	nd	nd	nd	0.010±0.001	0.011±0.001	0.025±0.001	0.012±0.001	0.046±0.002
benzyl alcohol	0.060±0.001	nd	0.084±0.002	nd	nd	0.029±0.001	nd	0.110±0.001	nd	0.211±0.001
3-octanol	nd	nd	nd	nd	0.021±0.003	0.017±0.002	0.039±0.003	0.024±0.002	0.050±0.008	nd
1-nonanol	nd	nd	nd	nd	nd	nd	0.005±0.001	0.015±0.001	nd	nd
phenethyl alcohol	nd	nd	nd	nd	nd	nd	0.094±0.002	0.284±0.003	0.240±0.002	0.484±0.003
1-octanol	nd	nd	nd	nd	0.002±0.001	nd	nd	0.002±0.001	nd	nd
2-ethyl-1-hexanol	nd	nd	nd	nd	0.002±0.001	nd	nd	nd	nd	nd
4-hepten-1-ol	nd	nd	nd	nd	nd	nd	nd	nd	nd	0.003±0.001
3-heptanol	nd	nd	nd	nd	nd	nd	nd	nd	nd	0.036±0.011
∑	0.06	0	0.084	0	0.025	0.056	0.149	0.461	0.302	0.807
**Esters**										
linalool acetate	0.018±0.001	nd	nd	nd	0.044±0.003	nd	nd	0.065±0.012	0.072±0.012	0.043±0.007
ethyl acetate	nd	1.254±0.012	0.468±0.031	0.483±0.002	0.511±0.032	0.636±0.011	0.133±0.021	0.221±0.003	0.131±0.001	0.201±0.002
ethyl butyrate	nd	nd	nd	nd	nd	nd	0.045±0.001	nd	nd	nd
ethyl isohexanoate	nd	nd	nd	nd	nd	nd	0.059±0.004	nd	nd	nd

ethyl valerate	nd	nd	nd	nd	nd	nd	0.082±0.002	nd	nd	nd
methyl (Z)-4-decenoate	nd	nd	nd	nd	nd	nd	nd	0.002±0.001	nd	nd
octyl isobutyrate	nd	nd	nd	nd	nd	nd	nd	0.002±0.001	nd	nd
linalyl butyrate	nd	0.002±0.001	0.002±0.001	nd	nd	nd	nd	nd	nd	nd
∑	0.018	1.256	0.47	0.483	0.555	0	0.319	0.29	0.203	0.244
**Terpenoids**										
1-octen-3-ol	nd	nd	0.054±0.001	0.004±0.001	0.012±0.001	0.017±0.001	0.024±0.001	0.019±0.002	0.024±0.001	0.020±0.001
linalool	0.364±0.012	0.657±0.009	0.275±0.007	0.627±0.011	0.340±0.007	0.619±0.021	0.412±0.020	0.581±0.005	0.509±0.008	0.528±0.042
α-terpineol	0.057±0.001	0.006±0.001	0.009±0.001	0.005±0.001	0.028±0.001	0.017±0.002	0.039±0.001	0.020±0.001	0.002±0.001	0.003±0.001
β-terpineol	nd	0.012±0.001	nd	nd	nd	0.022±0.003	nd	0.017±0.003	nd	nd
nerol	0.002±0.001	0.003±0.001	0.003±0.001	0.003±0.001	0.003±0.001	0.002±0.001	0.004±0.001	0.003±0.001	0.008±0.001	0.003±0.001
2,4-hexadien-1-ol	nd	nd	nd	nd	nd	0.002±0.001	nd	0.005±0.001	0.009±0.001	0.012±0.001
sabinene hydrate	0.016±0.002	nd	0.002±0.001	0.003±0.001	nd	nd	nd	nd	nd	nd
1,8-cineole	nd	0.011±0.001	nd	0.013±0.005	nd	0.022±0.001	nd	0.017±0.003	nd	0.023±0.003
geraniol	nd	nd	nd	nd	0.003±0.001	nd	0.003±0.001	nd	0.007±0.002	nd
myrcene	0.255±0.011	0.291±0.021	0.296±0.031	0.210±0.013	0.452±0.031	0.240±0.024	0.305±0.042	0.324±0.033	0.325±0.009	0.342±0.014
3-carene	0.008±0.001	0.004±0.001	0.005±0.001	0.006±0.001	0.003±0.001	0.004±0.001	0.006±0.001	nd	0.004±0.001	nd
α-phellandrene	0.021±0.004	0.032±0.005	0.007±0.002	0.059±0.004	0.097±0.003	0.013±0.004	0.006±0.001	0.028±0.004	0.030±0.003	0.092±0.003
β-phellandrene	0.005±0.001	nd	nd	nd	0.005±0.002	nd	nd	nd	0.012±0.003	nd
α-terpinene	0.089±0.012	0.017±0.003	0.079±0.002	0.123±0.019	0.107±0.013	0.116±0.032	0.084±0.005	0.256±0.021	0.157±0.025	0.218±0.009
limonene	nd	1.247±0.051	nd	0.847±0.021	nd	1.094±0.012	nd	0.908±0.031	nd	1.099±0.310
ocimene	0.133±0.012	0.126±0.013	0.177±0.034	0.643±0.045	0.199±0.017	0.287±0.021	0.096±0.011	0.384±0.014	0.496±0.043	0.532±0.051

terpinolene	0.005±0.001	nd	nd	nd	0.005±0.001	nd	0.003±0.001	nd	0.004±0.002	nd
allocimene B	0.026±0.021	0.038±0.004	0.086±0.003	0.018±0.012	0.016±0.004	0.007±0.001	0.008±0.002	0.006±0.001	0.017±0.003	0.006±0.002
β-caryophyllene	nd	0.002±0.001	0.002±0.001	nd	nd	0.007±0.001	0.004±0.001	nd	nd	nd
γ-terpinenes	0.026±0.011	0.064±0.032	0.039±0.031	0.054±0.012	0.009±0.002	0.014±0.002	0.004±0.001	0.016±0.002	0.003±0.001	0.014±0.002
isocaryophyllene	nd	nd	nd	nd	nd	0.002±0.001	nd	nd	nd	0.002±0.001
alloaromadendrene	nd	nd	nd	0.002±0.001	nd	nd	nd	nd	nd	nd
longifolene	nd	nd	nd	0.002±0.001	nd	nd	nd	0.002±0.001	nd	nd
β-humulene	nd	nd	nd	0.002±0.001	nd	nd	nd	nd	nd	nd
β-bourbonene	nd	nd	nd	0.002±0.001	nd	nd	0.002±0.001	nd	nd	nd
sabinene	nd	0.032±0.001	nd	0.022±0.001	nd	nd	0.021±0.003	nd	nd	nd
myrtenal	0.012±0.002	0.009±0.001	0.022±0.004	0.007±0.002	nd	nd	nd	nd	nd	nd
perilla ketone	nd	0.013±0.001	nd	0.042±0.001	nd	0.082±0.001	nd	0.102±0.011	nd	0.202±0.032
isothujone	0.087±0.021	nd	0.032±0.002	nd	0.052±0.003	0.006±0.002	0.085±0.006	0.012±0.021	0.072±0.001	0.078±0.003
α-thujone	nd	0.227±0.021	nd	0.234±0.021	nd	nd	nd	nd	nd	0.096±0.008
β-damascenone	nd	nd	nd	nd	nd	nd	nd	0.002±0.001	0.003±0.001	0.004±0.001
∑	1.106	2.791	1.088	2.928	1.331	2.573	1.106	2.702	1.682	3.274
**Aldehydes**										
2,4-dimethylbenzaldehyde	nd	nd	nd	nd	nd	nd	nd	nd	0.012±0.001	0.004±0.001
hexanal	nd	0.052±0.001	nd	0.042±0.001	0.009±0.001	nd	nd	0.032±0.001	nd	nd
heptanal	nd	0.002±0.001	nd	0.004±0.001	nd	nd	nd	0.006±0.001	nd	nd
∑	0	0.054	0	0.046	0.009	0	0	0.038	0.012	0.004
**Ketones**										

isophorone	nd	nd	nd	nd	nd	nd	nd	nd	nd	0.211±0.011
neryl acetone	0.003±0.001	0.004±0.001	0.003±0.001	nd	nd	nd	nd	0.002±0.001	nd	0.002±0.001
∑	0.090	0.244	0.035	0.265	0.052	0.088	0.085	0.118	0.075	0.593
**Sulfides**										
dimethyl trisulfide	nd	nd	nd	nd	nd	nd	0.178±0.007	0.075±0.005	0.243±0.002	0.017±0.004
dimethyl disulfide	nd	nd	nd	nd	nd	nd	0.584±0.028	0.398±0.028	0.932±0.088	0.432±0.034
∑	0	0	0	0	0	0	0.762	0.473	1.175	0.449
**Others**										
3-cyclohexen-1-ol	0.011±0.001	0.061±0.001	0.027±0.002	0.006±0.001	0.067±0.001	0.019±0.001	0.134±0.001	0.073±0.001	0.485±0.011	0.224±0.012
linalool oxide I	nd	nd	nd	nd	nd	nd	nd	0.002±0.001	nd	0.006±0.001
1-nonene	0.004±0.001	0.004±0.001	0.005±0.001	0.012±0.001	0.005±0.001	0.014±0.001	0.006±0.001	0.024±0.005	nd	nd
clovene	nd	nd	0.002±0.001	nd	nd	0.003±0.001	0.002±0.001	nd	nd	nd
2-bornene	nd	nd	nd	nd	nd	nd	nd	0.038±0.001	nd	nd
alanyl-L-alanine	nd	0.002±0.001	nd	nd	nd	nd	nd	nd	nd	nd
acetic acid	nd	nd	nd	nd	nd	nd	0.002±0.001	nd	0.102±0.021	nd
∑	0.015	0.067	0.034	0.018	0.072	0.036	0.144	0.137	0.587	0.23
Total content of volatile compounds	1.202	4.172	1.679	3.475	1.992	2.665	2.48	4.103	3.961	5.221

All values are the mean ± SD.
